# Feasibility of a mHealth Approach to Nutrition Counseling in an Appalachian State

**DOI:** 10.3390/jpm9040050

**Published:** 2019-11-20

**Authors:** Melissa D. Olfert, Makenzie L. Barr, Rebecca L. Hagedorn, Dustin M. Long, Treah S. Haggerty, Mathew Weimer, Joseph Golden, Mary Ann Maurer, Jill D. Cochran, Tracy Hendershot, Stacey L. Whanger, Jay D. Mason, Sally L. Hodder

**Affiliations:** 1 Department of Animal and Food Science, West Virginia University, Morgantown, WV 26505, USA; mbarr6@mix.wvu.edu (M.L.B.); rlhagedorn@mix.wvu.edu (R.L.H.); 2 School of Public Health, University of Alabama, Birmingham, AL 35487, USA; dmlong@uab.edu; 3 WVU Family Practice, Department of Family Medicine, Morgantown, WV 26501, USA; haggertyt@wvumedicine.org; 4 Valley Health System, Department of Family Medicine, Huntington, WV 25701, USA; mbweimer@valleyhealth.org; 5 New River Health, Department of Family Medicine, Sophia, WV 25921, USA; joseph.golden@nrhawv.org; 6 CAMC Family Practice, Department of Family Medicine, Charleston, WV 25304, USA; MAMAURER@hsc.wvu.edu; 7 Robert C. Byrd Clinic and West Virginia School of Osteopathic Medicine, Lewisburg, WV 24901 USA; jcochran@osteo.wvsom.edu; 8 Coplin Memorial Hospital, Family Practice, Elizabeth, WV 26143, USA; thendershot@wchsa.com; 9 West Virginia Practice Based Research Network, Morgantown, WV 26506, USA; swhanger@hsc.wvu.edu (S.L.W.); jdmason@hsc.wvu.edu (J.D.M.); 10 West Virginia University Health Sciences Center, Clinical and Translational Science Institute, Morgantown, WV 26506, USA; slhodder@hsc.wvu.edu

**Keywords:** rural, mHealth, feasibility, dietitian, app, Appalachia

## Abstract

West Virginia is a rural state with an aging population that may experience barriers to accessing nutritional and lifestyle counseling. This study examined feasibility of an online personalized nutrition tracking application, Good Measures (GM), with patients at seven health care clinics throughout the state. Fourteen healthcare providers and 64 patients 18 years or older with a Body Mass Index (BMI) greater than or equal to 30 and access to the Internet were recruited for this 12-week feasibility study. Patient participants logged meals and exercise into the GM application via smart phone, tablet, or computer and virtually engaged with a Registered Dietitian Nutritionist (RDN) in one-on-one sessions. The primary endpoint was to examine feasibility of the program by usage of the application and feedback questions regarding the benefits and challenges of the application. Participants were predominately white (92%) and female (76%). Minimal improvements in weight and systolic blood pressure were found. Participant attitude survey data declined from 4-weeks to 12-weeks of the intervention. Interestingly though, patients in a rural clinic had lesser declines in attitudes than peri-urban participants. Qualitative feedback data identified participants predominately had a positive overall feeling toward the approach. Participants expressed favorability of RDN access, the variety of foods, but did give suggestions for in-person meetings and more updating of the application. Implementing a technology approach to nutrition in rural areas of West Virginia using a mobile application with RDN access may be one strategy to address public health issues such as obesity.

## 1. Introduction

A significant portion of adults in the United States (US) have one or more nutritionally sensitive diseases including obesity, hypertension, hyperlipidemia, and diabetes mellitus [1,2,3,4,5,6]. Within the US, more than 78 million adults (34.9%) are obese, costing more than $200 billion annually [7,8]. The American Heart Association reports that one of every three Americans has hypertension with an estimated annual cost of nearly $50 billion [9,10,11,12,13]. Rates of obesity-related health issues in West Virginia are among the highest in the nation and have been so for decades [14]. Currently, West Virginia has the highest national prevalence of adults with obesity (38.1%), diabetes (15.2%), and hypertension (43.5%) [14]. Reduction of these co-morbidities has been at the forefront of strategic planning for improving lifestyles and quality of life.

Lifestyle factors, such as poverty and lack of education, have also been associated with obesity [15]. These factors, in addition to high mortality rates, have been predominately found among rural residents. Individuals living in rural areas are more likely to be obese than their urban counterparts, even after adjustment for poverty [16]. Lower-income individuals residing in rural areas often face barriers to acquiring high-quality diets and nutritional counseling including lack of transportation, long travel times, inability to pay, living in food deserts, and lack of understanding of quality food choices [17]. Individuals in a rural location have less opportunity to attend to preventative healthcare [18]. Travel distance for rural dwellers can impact the frequency at which they seek medical care. Healthcare providers in rural clinics have limited resources, increased workloads and longer hours when compared to their urban counterparts [19,20]. Concurrently, condition-specific or general dietary education is predominately initiated at visits with primary care healthcare providers although they may have inadequate training, time, or resources to provide nutrition counseling to patients [21]. In addition, medical professionals may lack nutritional training [22] and have been shown to know only as much about nutrition as those in the general public [19]. Nevertheless, even if weight and diet options were discussed with patients, they tend to have trouble recalling this face-to-face discussion with their health care provider [19]. An additional barrier to receiving nutrition services is a lack of insurance coverage for most nutritional counseling. This barrier likely results in patients lacking understanding of services provided by registered dietitian nutritionists (RDNs) and how to access those services.

Lifestyle interventions among persons with obesity-related co-morbidities can be beneficial; however, geographic challenges among rural persons may present unsurmountable barriers to receiving intervention services. Technology-based interventions via mobile phones or tablets can potentially overcome barriers to access [23,24]. Technology-based interventions have focused on multiple outcomes including depression, cardiovascular disease, weight loss and management, and engagement in risky behaviors with successful results [25,26,27,28,29,30,31]. Specifically, mobile technology-based health (mHealth) platforms are becoming an ideal methodology for technology-based interventions due to extensive growth and expected continued growth in mobile phone, particularly smart phone, use in the US [32]. Programs developed with an mHealth approach for weight management have shown to be feasible in the US and internationally [33,34,35,36]. Specifically for nutrition counseling, use of mHealth interventions are beneficial due to the increased ability of dietitians or nutritionists to provide tailored nutrition education for more individuals at lower cost than in-person counseling [37]. mHealth programming tailored towards nutrition education for weight loss has shown some promise for improving dietary patterns and weight status, although more testing is needed [38].

More importantly, the use of mHealth in rural communities has shown potential as a means to engage with hard-to-reach populations throughout rural America [25]. However, to our knowledge, only one study has tested the feasibility of using a mobile technology-based intervention in West Virginia [39]. This previous feasibility study tested the acceptability of an application to monitor and track chronic disease care in patients but was not specific to the lifestyle needs for weight loss such as access to an RDN. West Virginia is of particular importance due the aforementioned top rate of obesity among all states in the US States, thus the need for weight loss intervention. Further, 38% of West Virginians live in rural areas compared to 19.3% nationally, making West Virginians more prone to the barriers faced by rurality [40,41]. As no technology-based intervention has been implemented in the state for lifestyle changes, the perceptions of both physicians and patients to this approach is unclear. Therefore, investigating the feasibility of a technology-based intervention is warranted among this population.

The current feasibility study, titled Project Better Health (PBH), aims to test the acceptability of the smartphone/online application, Good Measures (GM), among overweight and obese men and women in rural West Virginia. The objectives are to: (1) assess attitudes toward GM and Smartphone Apps among patient participants, (2) examine frequency of GM app use by patient participants through week 4, (3) assess healthcare provider attitudes toward GM and Smartphone Apps. Post hoc analyses on rural and peri-urban populations will be completed to examine the acceptability in rural populations.

## 2. Materials and Methods 

### 2.1. Intervention Design

**Figure 1:**
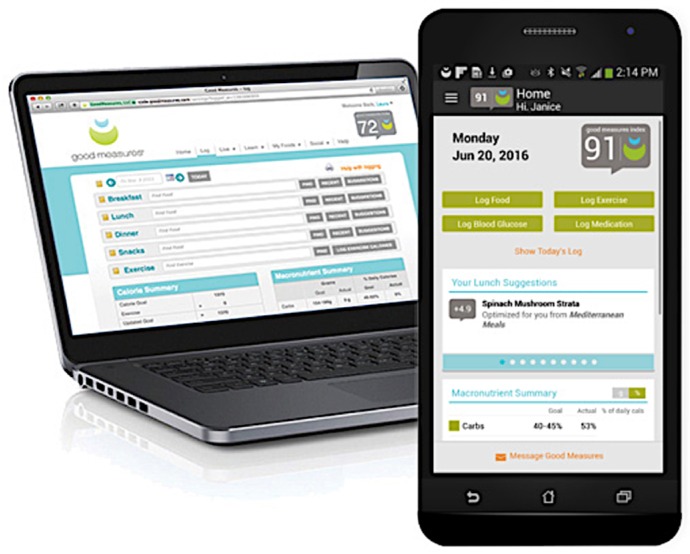
Good Measures Application shown in Mobile and Computer Format. Note: Written permission to use the Good Measures image is on file.

The GM platform used within this study was developed by Good Measures LLC (Good Measures, Boston, MA, USA, 2019). The GM platform, similar to other personalized applications that have consumers log their food, drink, and physical activity, heightens the current technology with one-on-one interactions with RDNs. The application platform is shown in Figure 1. This application combines the latest evidenced-based nutrition science with the power of a state-of-the-art digital platform (available via iPhone, Android, and the Web) to support healthy food choices and behavior change related to eating and exercise. With the GM platform, users can log their daily food intake and exercise and an RDN is able to track patient progress and communicate feedback through phone, email, Skype, or text at the patient’s discretion (frequency managed by participant). In addition, with every food entry into the application, the GM platform captures individuals eating patterns and calculates a “Good Measure Index (GMI),” an algorithm-derived number between 0–100 based on the nutritional balance of their dietary choices. The GM proprietary software provides real-time meal suggestions to help individuals meet nutritional goals by improving the GMI through “optimal” meal suggestions.

Prior to recruitment, clinic staff and healthcare providers were trained by research coordinators. Coordinators visited each clinic site for an 8 h training on the GM application, study procedures, and follow-up procedures. During training sessions, research staff trained all clinic staff on recruiting and enrolling participants, while GM staff assisted in the training of enrolled patients on use of the GM application. Participant training included clinic staff training on usage of GM, how to interact with RDNs, and an introduction to study questionnaires. Throughout the intervention, participants could seek assistance with the GM platform and were encouraged to utilize the application for the 12-week intervention.

### 2.2. Participants

Three levels of recruitment took place between September and November in 2015 for PBH including (1) clinical sites, (2) healthcare providers, and (3) patients. Inclusion criteria of study site healthcare providers were the access to a working smartphone or computer-based web applications, willing to complete study procedures including answer web-based questionnaires via a link from their email, and ability to read and comprehend English language. Patient inclusion criteria were: (1) Body Mass Index (BMI) greater than or equal to 30, (2) greater than 18 years, (3) access to the Internet, (4) willingness to complete questionnaires, and (5) English language comprehension. Patient exclusion criteria included actively participating in GM or another dietary program (e.g., PEIA Weight Management Program, Dean Ornish Prism Program, Nutrisystem, HMR, Weight Watchers, Jenny Craig), underlying medical condition with survival unlikely during follow-up period, any condition that would make participation in the study unsafe or interfere with achieving study objectives (e.g., mental illness), currently pregnant or breastfeeding, or if the patient was likely to move from the area in the next 4 months. Screening for eligibility into the program was performed initially by clinicians who identified patients eligible for enrollment.

Clinical sites were invited to participate through the West Virginia Practice-Based Research Network (WVPBRN), a catalyst for expanding research to rural healthcare facilities around the state. As part of the West Virginia Clinical Translational Science Institute’s (WVCTSI) Community Engagement and Outreach Core, the WVPBRN is currently comprised of 94 sites across the state, of which 7 were enrolled, based on clinic interest to participate in the GM study. From November 2015 to January 2016, healthcare providers and patients were recruited into the study. During that time, sites participated in weekly project calls to enhance site level support throughout the project. Of the groups recruited, all individuals (clinic staff, healthcare providers, and patients) received training on using the GM applications and completing all study procedures (informed consent, questionnaires, anthropometrics). West Virginia University Institutional Review Board approved this protocol #WVU02HSC2015-1508783458.

### 2.3. Study Design

Patient participants were tracked across 12 weeks to identify GM usage, GMI changes, attitudes toward the application, and anthropometric changes (weight and blood pressure). As this was a feasibility study looking at the acceptability and attitudes towards GM, no control group was recruited. Enrolled patients provided their written consent, were given access to the GM application, and were physically assessed at baseline and follow-up visits (4-weeks and 12-weeks) by their healthcare provider. Participants interaction with GM included a minimum of weekly engagement with the RDN via video call, phone call, email, or text message. Our two study RDNs were based out of the company headquarters in Boston, MA. Participants would additionally log their daily meals and snacks, drinks, and physical activity through the application. These daily logs assisted the RDN in understanding the patients and best areas for counseling and improvement. Patients had the autonomy to schedule their visits with the RDN at their discretion and space them out as needed. If patients failed to log into the app, RDNs would reach out a minimum of two times to encourage patients to re-engage.

### 2.4. Measures

At baseline clinic visits, participants completed informed consent documents and received anthropometric measures of height, weight and blood pressure by a trained staff member at each site in West Virginia. A 32-item patient behavioral and attitude survey was developed by the authors that assessed demographics, health history, technology capabilities, and attitudes toward a technology application. The full 32-item survey was completed at baseline. The 12 attitude questions were also administered mid-intervention (4-weeks), and post-intervention (12-weeks) to identify changes in attitudes toward the GM application. Attitudes toward the GM app were measured on a 9-point Likert item question with answers ranging from 1 being ‘Extremely Important” or ‘Strongly Agree’ to 9 being ‘Not Important’ or ‘Strongly Disagree’. Questions were also asked regarding the ease of using GM, its ease of understanding, if daily activities made it hard to log on the app, and if they would recommend GM to their family/friends/coworkers. Lastly, two open-ended questions asked participants to address the best/strongest part about GM, and to offer one or two items to improve GM.

Healthcare providers also completed questionnaires that included all questions in the form of answering in regard to their patient (i.e., “do you feel the GM app has helped your patients choose healthier foods?”). Finally, two open-ended questions were available for healthcare providers to provide feedback on positives and negatives of the GM platform. All surveys were administered via Qualtrics (Qualtrics, Provo, UT, USA), a secure, online survey platform.

Additionally, from the GMs data platform, usage of the application was calculated per user and averages were used. Available data compiled from the applications database included the number of meals logged, the number of exercise sessions logged, the total number of days logged, frequency of interaction with a dietitian, and GMI improvement. The number of meals logged in the application was used to determine usage and categorized as non-users (0–83 meals; less than 1 meal logged per week of study), low (84–167 meals), medium (168–251 meals), and high users (252 meals and above; three or more meals per day). From usage, percent weight improvement was examined from baseline weight to 12-week weight.

### 2.5. Statistical Methods

Baseline characteristics were compared using a Wilcoxon Rank Sum Test for continuous measures and Pearson Chi-Square for categorical variables with Fisher’s exact test for cell sizes <5. After completion of the intervention, as a post-hoc analysis, sites were broken into peri-urban and rural settings based on United States Department of Agriculture’s Rural-Urban Commuting Areas Codes (RUCA) [28]. GM application interaction and usage were analyzed by a Wilcoxon two-sample test for significance between the two geographical regions. Additionally, a Kruskal–Wallis test was used to test non-parametric percent weight improvement by app usage. Open-ended questions were analyzed using standard qualitative content analysis procedures [29,30]. Content analysis procedures were used as they produce unbiased, methodically derived descriptions of qualitative feedback [31,32]. Two trained researchers reviewed the data independently for themes, then discussed developed themes to come to a consensus between the two reviewers.

## 3. Results

### 3.1. Baseline Demographics

Sixty-four patient participants were consented and enrolled in the study (Table 1). The population was predominately white (90.8%), female (76.6%) and had a mean age of 44.9 ± 12.8 years (range of 19–63 years). Eighty-seven percent were taking prescribed medication and participant medical conditions included hypertension (43.8%), diabetes (26.6%), heart disease (7.8%), chronic obstructive pulmonary disease (COPD) (6.3%), cancer (1.6%), and others (17.2%). Participants largely used no tobacco (76.6%) and no alcohol (71.9%). Technology across the population indicated high internet capabilities (98.4%), high usage of smartphones (79.7%), and 43.8% using applications. Further, 23 participants had used a weight loss program in the past; highest usage with Weight Watchers (23.4%). Baseline weight of the population was an average of 256.8 ± 63.7 pounds, systolic blood pressure (SBP) of 128.8 ± 16.0 mmHg, and diastolic blood pressure (DBP) of 78.3 ± 11.0 mmHg. No harms or unintended consequences occurred during this study.

Out of the 64 participants that were enrolled, 56 continued to four weeks, and 50 continued to 12 weeks (78.1%). Of the population, our post hoc analysis examined peri-urban and rural site for differences. Of the participants, 26 came from a peri-urban site and 38 from a rural site. No significant difference was observed in demographics or retention between peri-urban and rural sites (84.6% vs. 73.7%, *p* = 0.36). Baseline weight and systolic blood pressure were not significantly associated with drop out at four weeks (*p* = 0.33, *p* = 0.34) or 12 weeks (*p* = 0.51, *p* = 0.77). While not significant, having a BMI > 35 at baseline increased the odds of dropping out by 50% (*p* = 0.63). There was no significant relationship between geographic region and drop-out (*p* = 0.85, *p* = 0.30).

### 3.2. GMs Application Usage

After study completion, GM analysts provided researchers with participant usage of the GM app to test feasibility of the application. Means and standard deviations are provided in Table 2. On average, the population logged 169.5 ± 155.1 meals, 25.3 ± 32.1 exercise sessions, and 55.3 ± 41.4 total days. Total RDN interaction for the population was 20.0 ± 17.0. Additionally, on the 0–100 GMI score, an average improvement of 12.0 ± 10.4 was found across the population at 12-weeks. As a post hoc analysis, peri-urban and rural sites were examined, and no significant differences were detected (Table 2). When breaking the population down by app engagement of meals logged, *n* = 22 were labeled as non-users (logging 0–83 meals total), *n* = 17 were low users (84–167 meals), *n* = 7 were medium users (168–251 meals logged), and *n* = 18 were high users (252 + meals logged). A Kruskal–Wallis test was used to examine percent weight improvement by app usage and detected significant differences by app usage group (Chi square = 7.48, *p* = 0.06, df = 3). Those high users of the application had average percent weight improvement of 0.02% ± 0.02%, medium user weight percent improvement average of 0.04% ± 0.04%, low user weight percent improvement average of 0.02% ± 0.03%, and non-user percent improvement average of 0.00% ± 0.02%. When comparing just those “users” vs. “non-users” there was a significant difference in percent weight improvement with our users average of 0.3% ± 0.3% and our non-users of 0.00% ± 0.02% (Chi-square = 6.56, *p* = 0.01, df = 1).

### 3.3. Attitude Measurements

Data are represented in average scores for rural and peri-urban settings between their 4-week and 12-week assessments (Table 3), with lower scores representing more positive attitudes. When examining attitudes toward the GM approach, overall attitudes declined, but by a miniscule amount. Post hoc analysis examined attitude changes between peri-urban and rural clinics. Peri-urban clinics had a decline in attitudes in eight of 10 variables, while rural clinics had a decline in attitudes in five of 10 categories. Peri-urban attitudinal improvements of scores included, (1) GM helped to choose healthier foods and (2) smartphone is a barrier to using the GM app. Declines in attitudes toward the app included all other questions except for rating that GM would help participants to choose healthier foods, which remained the same. In the rural population, improvements were seen in (1) nutrition being important to health, (2) GM increasing access to nutritional services, (3) internet being a barrier, (5) GM being easy to understand, and (6) wanting to use GM because of the way it was described.

From two open-ended questions, participants were asked to provide feedback on the areas of PBH and GM they enjoyed and areas that the program can improve. At the 12-week post-intervention point, 47 individuals completed the question regarding positives of the program and 34 answered the areas for improvement question. Themes and sample quotes are shown in Table 4. Of the positive areas, three major themes were found among the patient participants: (1) liked the access to the dietitian counseling on their own time (*n* = 20), (2) appreciated the range of food and meal options, and (3) valued calorie counting on the app (*n* = 20), and the overall app (*n* = 7). For the areas of improvement, three themes were found: (1) participants wanted more options of food and restaurants on the GM application (*n* = 7), (2) improvements of miscellaneous items such as monthly in-person meetings, (3) longer access to the intervention program, and more interactions, potentially in person (*n* = 12).

Attitudes from the healthcare provider surveys (*n* = 8) identified the training of the intervention and the GMs application as being beneficial, straightforward and adequate. They believed that motivated individuals utilized the application more frequently. Also, surveys indicated that patients may have enjoyed app use more if it could be used with their family or as a tool to motivate and communicate with others. Healthcare providers also felt that they had support from study staff when questions arose regarding the program. All providers indicated that they would like to work with the GM program in the future.

## 4. Discussion

This study aimed to test the feasibility of overweight or obese adults in West Virginia to engage in an interactive phone application, GM, to track their diet and physical activity. Patient and healthcare provider feedback from this feasibility study show promise for the use of a mobile technology-based intervention among overweight and obese patients in rural West Virginia. 

Among patients, nutrition was seen as an important factor towards health and GM was seen as a means to helping patients choose healthy foods. However, regarding attitudes toward the GM application, 80% of peri-urban categories had a decline in other attitudes, while 50% of categories declined in the rural population. The decline in some attitudes may highlight barriers participants faced including the use and understandability of the GM application. Of particular interest though are the more favorable views from rural participants. Specifically seeing this interest in an mHealth approach from patients of a rural clinic is of interest for future technology-based interventions in hard-to-reach, or health-disparate, populations. Previous research has shown that mHealth approaches in rural and underdeveloped areas can be effective at improving lifestyle behaviors. For example, a systematic review of mHealth approaches in developing countries reported that 50% of physical activity and 70% of dietary interventions were deemed effective [42]. Further, decades of research on attitudes impact on behavior change has shown that positive attitudes towards change are more likely to result in positive outcomes [43]. Thus, this finding may highlight that use of a mHealth application can be effective in behavior change in more rural, underdeveloped populations.

Further, although we saw average declines in some attitudinal survey ratings, two open-ended questions prompted participants to describe the positives and negatives of the application and program. These descriptions brought to light positive aspects of the application and program that the quantitative questions could not capture. Positive feedback included use of the RDN, variety of foods available on the app, and general quality of the intervention overall. As this was targeted as a feasibility study, these descriptions highlight the usefulness of the program and the potential for future use. Similar to Mallow et al.’s study in a West Virginian population, increased access and communication with the healthcare provider, in this case an RDN, was seen as a positive component of the mHealth platform [39]. This finding of desire for personalized RDN counseling and one-on-one sessions was encouraging and similar to other rural technology-based approaches, suggesting the importance of using a personalized approach to nutrition care [44]. Previous studies show the unique capabilities of mHealth intervention approaches and their usefulness in enhancing the health of community members [45]. Studies have also shown that these approaches are important to rural community members. In an intervention targeting pharmacy use through mHealth, participants stated that the approach was useful in their efforts to improve lifestyle [46].

From open-ended feedback questions, participants predominately praised the application method for their lifestyle and specifically highlighted the appreciation for access to an RDN. Some negative comments highlighted the lack of certain restaurants and foods on the app, and there was some suggestion that further interaction such as in-person monthly meetings would be useful. Potentially incorporating group messaging or discussion boards, group video conferences, or application competitions may enhance a sense of camaraderie and interest in the application and program overall. A study examining a multilevel intervention guided through an ecologic theory model framework to enhance physical activity of adults found that text messaging, support walking groups, and community events were all used to enhance activity [47]. Employing a future model—also guided by ideas mentioned by participants in this study—that uses additional levels of influence could be useful for mHealth interventions.

As with population studies, authors understand that not all participants will engage to the fullest capacity with the application. Although study participants positively reviewed this program, we also noticed this limited usage of meals logged on the app. With full usage of the application, by calculating an average participant logging of at least three main meals throughout each day of the twelve weeks, total meals logged would’ve equaled 252 meals. When looking at our outcomes, we identified that our participants were logging an average of about 90 meals less than this expected example. In a similar previous study with an obese adolescent population utilizing Fitbit technology, they found no changes in weight, a decrease in the usage of the Fitbit technology, and that the excitement of the program was short-lived [48]. Although we did find a limited amount of meals logged on the app total, this is common within mHealth studies as multiple feasibility studies in the US and internationally have shown high-dropout and low-participation rates [33,36]. From this lack of interaction, it can be delineated that the GM platform should include reminders to log all meals in an effort to facilitate more complete data entry. 

As mHealth platforms become more popular, it is suggested that health professionals globally be trained to engage with and implement mobile platforms into their practice [49,50,51,52]. Specifically, for nutrition counseling, Chen et al. identified only 24% of surveyed dietitians part of the Australian, New Zealand or British dietetic associations had completed professional development on how to use and implement mHealth applications for behavior change [49]. This may lead to health care professionals who are not adequately equipped to address barriers patients face while using a mobile platform. Further, the sustainability of these programs may be limited by barriers patients face for insurance coverage of mHealth [53,54], thus health care professionals should advocate for more insurance coverage for mHealth programming. While participants in this intervention received access to GM free of cost, in a real-world setting this is not the case. GM services, along with many other mHealth applications, require reimbursement that is not always covered by insurance, limiting the sustainability of use for the general population. Therefore, advocating for health policy changes to ensure stronger insurance coverage of the mobile applications, such as GM, could help promote more sustained use.

Although we did find positive outcomes in attitudes, this study was not without limitations. As this was a feasibility study, the small sample size precludes generalization of results without a larger study. Our population was predominantly white and female with the ability to utilize computers and the internet, hence our results may not apply to other populations. Rural populations may lack broadband and smartphone availability, limiting the accessibility of this approach, although this feasibility study provides promise as many rural participants did not view smartphone or internet use as a barrier. Further, as a feasibility study, no control group was used. The lack of control group limited researchers from being able to track the success of GM for weight loss outcomes. However, prior to studying the impact of GM, it was necessary to understand if this West Virginian population was open to engaging in this type of intervention as no previous research has addressed this issue. This feasibility study serves as an initial step that provides promise for the success of a larger intervention study in this population. The study duration was also brief (12 weeks) but similar to the previous study by Mallow et al. [39].Therefore, this feasibility design is unable to capture sustainable, long-term, behavior change along with medication changes or disease remission. Additionally, it should be noted that the intervention timeframe encompassed some major holiday seasons that include larger meal gatherings, which could influence the compliance or engagement with the application. When we examine the engagement of participants with the application, we find a large variability in the meals, exercise, days, and RDN interactions logged, which could potentially be attributed to motivation during the holiday season. A larger longitudinal study with this application among a more diverse study population is warranted.

## 5. Conclusions

Findings from the current study indicate that a mobile health approach to healthier nutrition and lifestyles is feasible for rural populations within West Virginia. Attitudes toward the GM application were positive and indicate potential future use of the model. Participants across West Virginia found the approach to be helpful, and rural individuals had positive attitudes toward the application and usage. Use of mobile device platforms, such as GMs, may broaden availability of nutritional and lifestyle coaching to rural populations though further research is needed to better understand optimization of this approach.

## Figures and Tables

**Table 1:** Population demographics.

Variable	Total
	(*n* = 64)
*Demographic*	
Age (years)	44.9
Gender	
Male	15
Female	49
Race/Ethnicity	
White only	59
Other (including black only, Asian only, and bi-racial)	6
Geography	
Peri-urban	26
Rural	38
Co-morbidities	
Diabetes	17
Hypertension	28
Heart Disease	5
Cancer	1
COPD	4
Sleep apnea	9
Other	11
Taking prescribed medication	
Yes	56
No	9
Technology capabilities	
Internet	63
Smartphone	51
Use Apps	28
*Anthropometric*	
Weight (lbs)	256.8 ± 63.7
Systolic Blood Pressure (mmHg)	128.8 ± 16.0
Diastolic Blood Pressure (mmHg)	78.3 ± 11.0

COPD, chronic obstructive pulmonary disease.

**Table 2:** Good Measures (GM) application usage.

	Total	Peri-Urban	Rural
	*n* = 64	*n* = 26	*n* = 38
Number of Meals Logged	169.5 ± 155.1	172.5 ± 142.3	167.5 ± 165.1
Number of Exercise Sessions Logged	25.3 ± 32.1	23.4 ± 29.4	26.6 ± 34.2
Number of Days logged	55.3 ± 41.4	57.8 ± 32.6	53.5 ± 46.8
Total RDN Interactions	20.0 ± 17.0	21.6 ± 17.4	18.9 ± 16.8
GMI Improvement	12.0 ± 10.4	10.0 ± 7.8	13.7 ± 12.2

Variables collected from GMs interface data and presented in means and standard deviations. No significant differences detected.

**Table 3:** Attitude and behaviors of the population toward Good Measures.

	Total (*n =* 64)		Peri-Urban (*n =* 26)		Rural (*n =* 38)	
Variable	4	12	4	12	4	12
Nutrition important for health	2.27	2.08	2.68	2.71 *	1.96	1.61
GM increase access to nutritional services	2.18	2.29 *	2.43	2.82 *	2.00	1.90
GM helps to reach goals	2.25	3.45 *	2.61	4.10 *	2.00	2.97 *
GM helps to choose healthy food	2.40	2.46 *	2.74	2.73	2.16	2.25 *
A smartphone is a barrier to using GM	6.67	6.41 *	7.35	7.73	6.19	6.17 *
The Internet is a barrier to using GM	6.53	6.68	7.70	7.18 *	5.69	6.29
GM is easy to understand	2.15	2.25 *	2.23	2.95 *	2.10	1.72
GM description made me want to use it	2.26	2.41 *	2.52	3.23 *	2.10	1.79
Daily activities prevent me from using GM	5.62	5.06 *	5.43	4.68 *	5.75	5.34 *
I would recommend GM	2.05	2.31 *	2.39	2.95 *	1.81	1.83 *

* Denotes a decline in attitudinal rating averages from week 4 to week 12.

**Table 4:** Qualitative themes with associated quotes from participants.

Question Topic	Themes	Related Quotes
Positives of the program	1.1 Access to the dietitian 1.2 Range of food and meal options 1.3 Calorie counting	“The ability to track calories and food intake, you don’t realize how much you really take in but when you see it in front of your eyes, it’s enlightening.” “Logging my food keeps me mindful of what I intend to eat, knowing I have to write it down. I also enjoy having discussions with my dietitian.” “It makes you aware of how much junk you consume in a day, I have made better choices and have lost 12 lbs.” “I love being able to speak to my dietitian any time I need.” “I benefit from the availability of the Good Measures Index (GMI). like that I am able to see what nutrients I am missing or that I have too much of. The allows me to cater my diet and future meals to meet the goals.”
2 Room for Improvement	2.1 More food and restaurant options 2.2 Need for in-person meetings 2.3 Longer program access	“Better relation to WV fast food. I have to get online and find the nutritional value of some of the WV unique fast food.” “Have more local brands of foods from the area that this is populated towards.” “I recommend having some monthly meetings in person with the clients.” “I wish it was longer access.” “I think it would be good to have monthly meetings and some kind of exercise class.”

## Supplementary Files

Supplementary File 1

## References

[1] Sacks F.M., Carey V.J., Anderson C.A., Miller E.R., Copeland T., Charleston J., Harshfield B.J., Laranjo N., McCarron P., Swain J. (2014). Effects of high vs low glycemic index of dietary carbohydrate on cardiovascular disease risk factors and insulin sensitivity: The OmniCarb randomized clinical trial. JAMA.

[2] Pilic L., Pedlar C.R., Mavrommatis Y. (2016). Salt-sensitive hypertension: Mechanisms and effects of dietary and other lifestyle factors. Nutr. Rev..

[3] Estruch R., Ros E., Salas-Salvadó J., Covas M.-I., Corella D., Arós F., Gómez-Gracia E., Ruiz-Gutiérrez V., Fiol M., Lapetra J. (2018). Primary prevention of cardiovascular disease with a Mediterranean diet supplemented with extra-virgin olive oil or nuts. N. Engl. J. Med..

[4] Ando K., Matsui H., Fujita M., Fujita T. (2010). Protective effect of dietary potassium against cardiovascular damage in salt-sensitive hypertension: Possible role of its antioxidant action. Curr. Vasc. Pharmacol..

[5] Kaczmarczyk M.M., Miller M.J., Freund G.G. (2012). The health benefits of dietary fiber: Beyond the usual suspects of type 2 diabetes mellitus, cardiovascular disease and colon cancer. Metabolism.

[6] Sun Y., You W., Almeida F., Estabrooks P., Davy B. (2017). The effectiveness and cost of lifestyle interventions including nutrition education for diabetes prevention: A systematic review and meta-analysis. J. Acad. Nutr. Diet..

[7] Spieker E.A., Pyzocha N. (2016). Economic impact of obesity. Prim. Care Clin. Off. Pract..

[8] Tremmel M., Gerdtham U.-G., Nilsson P., Saha S. (2017). Economic burden of obesity: A systematic literature review. Int. J. Environ. Res. Public Health.

[9] Zhang D., Wang G., Zhang P., Fang J., Ayala C. (2017). Medical expenditures associated with hypertension in the US, 2000–2013. Am. J. Prev. Med..

[10] Yoon S.S., Burt V., Louis T., Carroll M.D. (2012). Hypertension among adults in the United States, 2009–2010. NCHS Data Brief.

[11] Arrieta A., Qiao N., Woods J.R., Jay S.J., Veledar E., Nasir K. (2015). Cost of Cardiovascular Disease Episodes among Patients with Hypertension. Circ. Cardiovasc. Qual. Outcomes.

[12] Benjamin E.J., Muntner P., Bittencourt M.S. (2019). Heart disease and stroke statistics-2019 update: A report from the American Heart Association. Circulation.

[13] Moran A.E., Odden M.C., Thanataveerat A., Tzong K.Y., Rasmussen P.W., Guzman D., Williams L., Bibbins-Domingo K., Coxson P.G., Goldman L. (2015). Cost-effectiveness of hypertension therapy according to 2014 guidelines. N. Engl. J. Med..

[14] The State of Obesity in West Virginia. https://www.stateofobesity.org/states/wv/.

[15] Levine J.A. (2011). Poverty and obesity in the US. Am. Diabetes Assoc..

[16] Befort C.A., Nazir N., Perri M.G. (2012). Prevalence of obesity among adults from rural and urban areas of the United States: Findings from NHANES (2005–2008). J. Rural Health.

[17] Garcia M.C., Faul M., Massetti G., Thomas C.C., Hong Y., Bauer U.E., Iademarco M.F. (2017). Reducing potentially excess deaths from the five leading causes of death in the rural United States. MMWR Surveill. Summ..

[18] Chan L., Hart L.G., Goodman D.C. (2006). Geographic access to health care for rural Medicare beneficiaries. J. Rural Health.

[19] Haggerty T., Xiang J., Doyle G. (2016). Patient Attitudes toward Weight Related Discussions in Rural Appalachian Primary Care Clinics. W. Va. Med. J. OA.

[20] Kushner R.F. (1995). Barriers t o providing nutrition counseling by physicians: A survey of primary care practitioners. Prev. Med..

[21] Parker W.-A., Steyn N.P., Levitt N.S., Lombard C.J. (2011). They think they know but do they? Misalignment of perceptions of lifestyle modification knowledge among health professionals. Public Health Nutr..

[22] Barratt J. (2001). Diet-related knowledge, beliefs and actions of health professionals compared with the general population: An investigation in a community Trust. J. Hum. Nutr. Diet..

[23] Anderson-Lewis C., Darville G., Mercado R.E., Howell S., Di Maggio S. (2018). mHealth technology use and implications in historically underserved and minority populations in the United States: Systematic literature review. JMIR mHealth uHealth.

[24] Miyamoto S.W., Henderson S., Young H.M., Pande A., Han J.J. (2016). Tracking health data is not enough: A qualitative exploration of the role of healthcare partnerships and mHealth technology to promote physical activity and to sustain behavior change. JMIR mHealth uHealth.

[25] Cluxton-Keller F., Buteau J., Williams M., Stolte P., Monroe-Cassel M., Bruce M. (2019). Engaging rural young mothers in a technology-based intervention for depression. Child Youth Serv..

[26] Murry V.M., Berkel C., Inniss-Thompson M.N., Debreaux M.L. (2019). Pathways for African American Success: Results of Three-Arm Randomized Trial to Test the Effects of Technology-Based Delivery for Rural African American Families. J. Pediatr. Psychol..

[27] Dallery J., Raiff B.R., Grabinski M.J., Marsch L.A. (2019). Technology-Based Contingency Management in the Treatment of Substance-Use Disorders. Perspect. Behav. Sci..

[28] Gore M.O., Krantz M.J., Albright K., Beaty B., Coronel-Mockler S., Bull S., Estacio R.O. (2019). A controlled trial of mobile short message service among participants in a rural cardiovascular disease prevention program. Prev. Med. Rep..

[29] Hageman P.A., Mroz J.E., Yoerger M.A., Pullen C.H. (2019). User Engagement Associated with Web-Intervention Features to Attain Clinically Meaningful Weight Loss and Weight Maintenance in Rural Women. J. Obes..

[30] Khan K.M., Evans S.S., Bielko S.L., Rohlman D.S. (2018). Efficacy of technology-based interventions to increase the use of hearing protections among adolescent farmworkers. Int. J. Audiol..

[31] Downs D.S., Smyth J.M., Heron K.E., Feinberg M.E., Hillemeier M., Materia F.T. (2019). Beliefs about Using Smartphones for Health Behavior Change: An Elicitation Study with Overweight and Obese Rural Women. J. Technol. Behav. Sci..

[32] Dicianno B.E., Parmanto B., Fairman A.D., Crytzer T.M., Yu D.X., Pramana G., Coughenour D., Petrazzi A.A. (2015). Perspectives on the evolution of mobile (mHealth) technologies and application to rehabilitation. Phys. Ther..

[33] Hebden L., Cook A., Van der Ploeg H., King L., Bauman A., Allman-Farinelli M. (2014). A mobile health intervention for weight management among young adults: A pilot randomised controlled trial. J. Hum. Nutr. Dietetics.

[34] Gan K.O., Allman-Farinelli M. (2011). A scientific audit of smartphone applications for the management of obesity. Aust. N. Z. J. Public Health.

[35] Waterlander W., Whittaker R., McRobbie H., Dorey E., Ball K., Maddison R., Smith K.M., Crawford D., Jiang Y., Gu Y. (2014). Development of an evidence-based mHealth weight management program using a formative research process. JMIR mHealth uHealth.

[36] Mhurchu C.N., Whittaker R., McRobbie H., Ball K., Crawford D., Michie J., Jiang Y., Maddison R., Waterlander W., Myers K. (2014). Feasibility, acceptability and potential effectiveness of a mobile health (mHealth) weight management programme for New Zealand adults. BMC Obes..

[37] Probst Y., Nguyen D.T., Rollo M., Li W. (2014). mHealth diet and nutrition guidance. mHealth Multidisciplinary Verticals.

[38] McCarroll R., Eyles H., Mhurchu C.N. (2017). Effectiveness of mobile health (mHealth) interventions for promoting healthy eating in adults: A systematic review. Prev. Med..

[39] Mallow J.A., Theeke L.A., Walls R., Theeke E., Mallow B.K. (2016). Part B: The feasibility and acceptability of mI SMART, a nurse-led technology intervention for multiple chronic conditions. Open J. Nurs..

[40] What is Rural America. https://www.census.gov/library/stories/2017/08/rural-america.html.

[41] Service E.R. State Fact Sheets: West Virginia. https://data.ers.usda.gov/reports.aspx?StateFIPS=54&StateName=West%20Virginia&ID=17854.

[42] Müller A.M., Alley S., Schoeppe S., Vandelanotte C. (2016). The effectiveness of e-& mHealth interventions to promote physical activity and healthy diets in developing countries: A systematic review. Int. J. Behav. Nutr. Phys. Act..

[43] Sheeran P., Maki A., Montanaro E., Avishai-Yitshak A., Bryan A., Klein W.M., Miles E., Rothman A.J. (2016). The impact of changing attitudes, norms, and self-efficacy on health-related intentions and behavior: A meta-analysis. Health Psychol..

[44] Bailey J., Davies C., McCrossin T., Kiernan M., Skinner R., Steinbeck K., Mendis K. (2018). Fit4YAMs: Structuring a lifestyle intervention for rural overweight and obese young adult males using participatory design. J. Adolesc. Health.

[45] Martin T. (2012). Assessing mHealth: Opportunities and barriers to patient engagement. J. Health Care Poor Underserved.

[46] Sankaranarayanan J., Sallach R.E. (2014). Rural patients’ access to mobile phones and willingness to receive mobile phone-based pharmacy and other health technology services: A pilot study. Telemed. e-Health.

[47] Beck A.M., Eyler A.A., Hipp J.A., King A.C., Tabak R.G., Yan Y., Reis R.S., Duncan D.D., Gilbert A.S., Serrano N.H. (2019). A multilevel approach for promoting physical activity in rural communities: A cluster randomized controlled trial. BMC Public Health.

[48] Yoost J., Gerlach J., Sinning K., Cyphert H. (2018). The Use of Fitbit Technology Among Rural Obese Adolescents. J. Obes. Nutr. Disord..

[49] Chen J., Lieffers J., Bauman A., Hanning R., Allman-Farinelli M. (2017). The use of smartphone health apps and other mobile health (mHealth) technologies in dietetic practice: A three country study. J. Hum. Nutr. Diet..

[50] Gagnon M.-P., Ngangue P., Payne-Gagnon J., Desmartis M. (2015). m-Health adoption by healthcare professionals: A systematic review. J. Am. Med. Inform. Assoc..

[51] O’Donovan J., Bersin A., O’Donovan C. (2015). The effectiveness of mobile health (mHealth) technologies to train healthcare professionals in developing countries: A review of the literature. BMJ Innov..

[52] Chen J., Gemming L., Hanning R., Allman-Farinelli M. (2018). Smartphone apps and the nutrition care process: Current perspectives and future considerations. Patient Educ. Couns..

[53] Weinstein R.S., Lopez A.M., Joseph B.A., Erps K.A., Holcomb M., Barker G.P., Krupinski E.A. (2014). Telemedicine, telehealth, and mobile health applications that work: Opportunities and barriers. Am. J. Med..

[54] Vidmar A.P., Salvy S.J., Pretlow R., Mittelman S.D., Wee C.P., Fink C., Fox D.S., Raymond J.K. (2019). An addiction-based mobile health weight loss intervention: Protocol of a randomized controlled trial. Contemp. Clin. Trials.

